# A case report: anteroseptal ST elevation due to acute isolated right ventricular infarction

**DOI:** 10.1186/s12245-023-00522-z

**Published:** 2023-07-28

**Authors:** Indah Sukmawati, Fang Qin Goh, Alfred Yip, Poay Huan Loh, Koo Hui Chan

**Affiliations:** 1 National University Heart Centre Singapore, 5 Lower Kent Ridge Road, Singapore, 119074 Singapore; 2 Siloam Hospitals Lippo Village Indonesia, Jalan Siloam No 6, Tangerang, 15811 Indonesia; 3 National University Hospital, 5 Lower Kent Ridge Road, Singapore, 119074 Singapore

**Keywords:** Electrocardiogram, STEMI, RV myocardial Infarction

## Abstract

**Background:**

Electrocardiogram (ECG) is the first diagnostic tool physicians use in diagnosing acute myocardial infarction (MI). In this case report, we present a case where the initial ECG diagnosis was that of an acute anteroseptal MI but emergency coronary angiography showed that the infarct-related artery was a small non-dominant right coronary artery (RCA) instead of the anticipated left anterior descending artery (LAD). Isolated right ventricular (RV) infarction from a non-dominant RCA is rarely seen in clinical practice, and it may exhibit ECG changes that can be confused with an acute anteroseptal MI. It is important to appreciate the subtle differences in the ECG changes that occur in either of these two types of MI for appropriate diagnosis and treatment.

**Case presentation:**

A 49-year-old non-smoking male with prior coronary stent implantation in LAD presented with acute chest pain and his pre-hospital ECG indicated an anteroseptal STEMI possibly due to stent thrombosis, but an emergency angiogram showed patent LAD and Circumflex arteries. There was however thrombotic occlusion of the right, non-dominant coronary artery, which was revascularized with a drug-eluting stent. The patient’s chest pain and ST elevations resolved, and subsequent echo showed moderate RV systolic dysfunction in keeping with RV myocardial infarction.

**Discussion:**

RV myocardial infarction is usually due to an occlusion of the dominant RCA proximal to the origin of its RV wall branch, which often results in inferior ST elevation with reciprocal anterior ST depression. The ST elevation over V1 which would accompany RV infarction is often masked due to the more dominant electrical forces of inferior and posterior LV wall infarction. Our case demonstrates that in isolated RV infarction due to non-dominant proximal RCA occlusion, anterior ST elevation can be seen over V1-3, being most prominent in V1, which overlies the right ventricle, and resolved after restoring flow to the RCA. Spatial vector analysis of the ECG or right-sided ECG leads would be helpful to aid the diagnosis of RV infarction when clinical suspicion is present, for example when there is significant hypotension, raised jugular venous pressure but clear lung fields or deterioration after nitrate administration.

## Background

The electrocardiogram (ECG) is an essential tool used by physicians in the diagnosis of acute myocardial infarction. In this case report, we describe a patient presenting with acute chest pain whose ECG was initially interpreted to show signs of an anteroseptal MI but his emergency ccoronary angiogram showed that the culprit lesion was actually in the right coronary artery (RCA) which was a small, non-dominant vessel. We highlight the importance of careful interpretation of ECG findings to avoid delays in making the right diagnosis and to understand subtle clues to correctly identify the culprit lesion for revascularisation in patients with suspected acute coronary syndrome.

## Case presentation

**Fig. 1:**
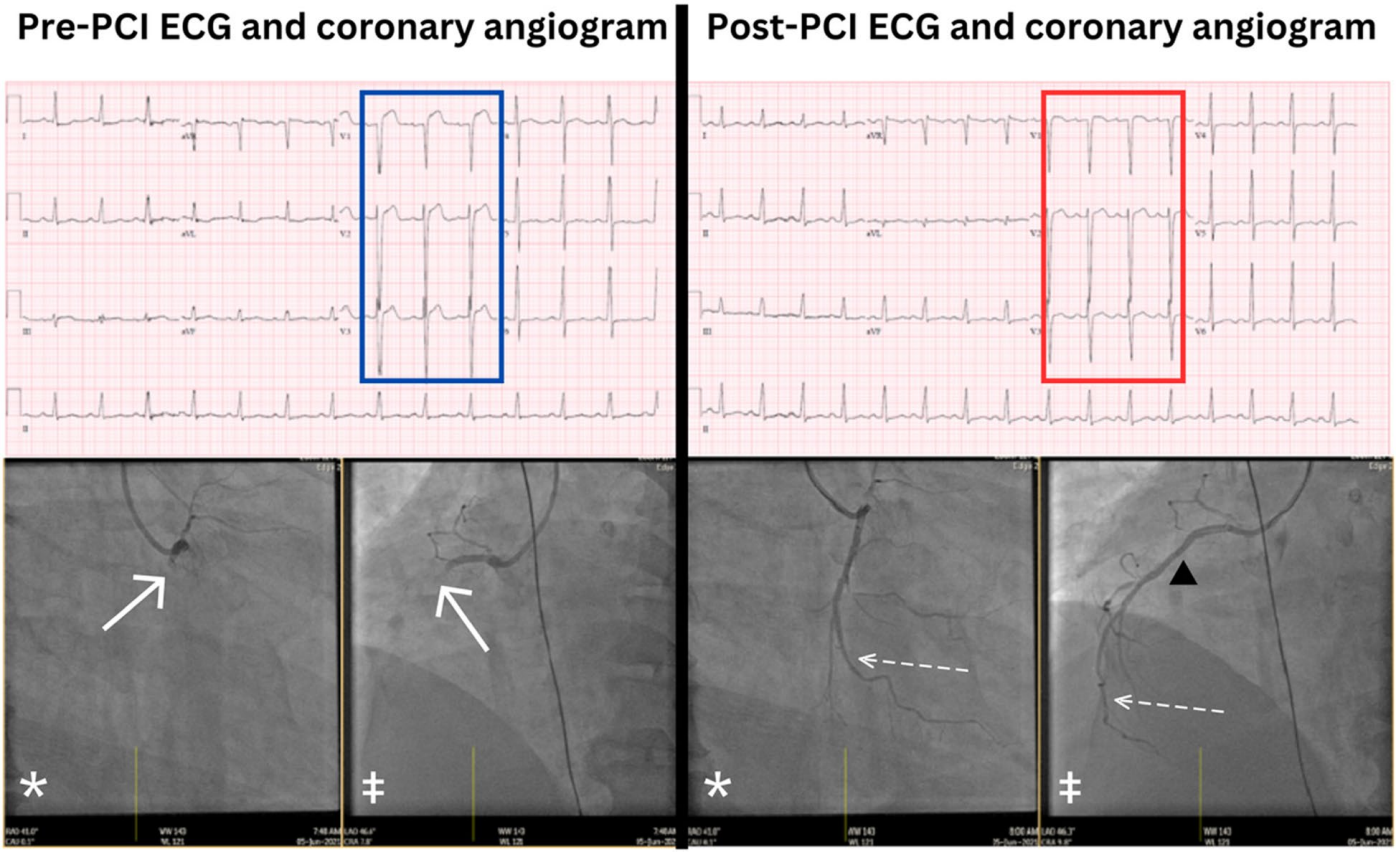
Anterior ST elevations over V1–3 in the pre-PCI ECG (blue box) due to proximal occlusion in a non-dominant RCA (solid arrows→) on the coronary angiogram (right anterior oblique view (*) and left anterior oblique view (‡). The ECG changes (red box) resolved after a stent (▲) was deployed, restoring flow to the RV wall branch (dashed arrows –- >)

A 49-year-old male non-smoker with prior coronary stent implantation to his proximal left anterior descending artery (LAD) in 2015, presented with an hour history of chest discomfort radiating to his jaw, nausea, and diaphoresis. The pre-hospital ECG demonstrated ST elevation over leads V1-3, (most marked in V1, Fig. 1), and the cardiac catheterization laboratory was activated for an anteroseptal ST elevation myocardial infarction (STEMI), possibly due to very late stent thrombosis in view of his previous cardiac history. He was hemodynamically stable with no clinical signs of heart failure. He was given intravenous morphine for analgesia.

Emergency coronary angiogram however demonstrated a left dominant coronary circulation with patent LAD and Circumflex arteries although there was significant in-stent restenosis in the proximal LAD which appeared stable angiographically with normal antegrade flow. There was thrombotic occlusion of the right coronary artery proximally, and this was deemed the culprit lesion and revascularized with the implantation of a drug-eluting stent which restored normal flow distally including the right ventricular (RV) wall branch (Fig. 1, dashed arrows). This resulted in the resolution of his chest pain and ST elevation. His subsequent ECGs post-reperfusion (Fig. 1) did not show any evolutionary *Q* waves or *T* wave inversion in the anteroseptal leads. Pre-discharge trans-thoracic echocardiogram showed normal RV chamber size, but there was moderate RV systolic dysfunction in keeping with RV myocardial infarction.

## Discussion

Our case demonstrates that RV myocardial infarction (RVMI) can present with precordial ST elevation. The RCA perfuses the RV via its RV wall branch. The dominant RCA typically supplies the inferior and posterior left ventricular (LV) wall whereas the non-dominant RCA supplies blood to just the RV and not the LV myocardium. RVMI can occur due to RCA occlusion proximal to the RV wall branch or occlusion of the RV wall branch itself which may sometimes occur after mid-RCA stenting when the RV branch is jailed by the stent [1].

RVMI occurs in approximately 30 to 50% of inferior MI due to occlusion of the proximal RCA [2]. The V1 lead lies over the right ventricle, and ST elevation over V1 that accompanies RVMI is often masked during an inferior STEMI, due to the more dominant electrical forces (from larger myocardial mass) associated with inferior and posterior LV wall infarction [3, 4] —the concurrent reciprocal anterior ST depression negates the ST elevation in V1. In view of this, only 7% of all MIs with precordial ST elevation had been attributed to occluded RCA/RVMI [5]. Inferior MI with concurrent RVMI due to proximal occlusion of a dominant RCA can exhibit precordial ST elevation if there is RV dilatation, but there have been cases also reported to show precordial ST elevation without RV dilatation [6], for example, if the dominant RCA is a small vessel whose injury current is not large enough to counteract the RV injury current.

Proximal occlusion of a non-dominant RCA causing an isolated RVMI can also give rise to precordial ST elevation as demonstrated in our case. Isolated RVMI is very rare and occurs in less than 3% of all patients with MI in an autopsy series [7]. A PubMed database search for cases of isolated right ventricular infarction published between 2000 and 2021 resulted in 19 reports [8]. For our case, the initial impression of the ST elevation in leads V1–V3 on the admission ECG was that of an acute anteroseptal MI. However, careful inspection and recognition of several typical features of isolated RVMI present on our patient’s ECG would have helped to avoid such misinterpretation. In isolated RVMI, the ST elevation has a dome-like appearance with a decremental amplitude from V1 to V3. Upon reperfusion and recovery, the ST elevation resolved rapidly with neither evolutionary *T* wave changes nor *Q* wave formation in the affected leads [4]. Often a right-sided ECG is done in cases of suspected RV infarction, as ST elevation in V4R has a high sensitivity and specificity for RV infarction [9], but our patient’s ECG showed ST depression in lead I indicating a rightward mean spatial ST segment vector in the frontal plane and in combination with an anteriorly directed mean spatial ST segment vector in the horizontal plane, suggested an epicardial injury in the RV [10].

Although the admission ECG for our patient was initially misinterpreted, this did not result in any adverse impact on his clinical management. There was no delay in the patient receiving his timely reperfusion therapy, namely primary PCI, as he had ST elevation on his ECG. The patient was given IV morphine and not nitrate therapy for his chest pain although in the Cath lab, he received intra-arterial nitroglycerin after inserting a vascular sheath in his right radial artery. He was hemodynamically stable throughout the procedure. The correct culprit lesion was correctly identified even though there was also significant restenosis in his previous proximal LAD stent. It is important to distinguish correctly the precordial ST elevation due to anterior MI and RVMI as a misdiagnosis can potentially result in the administration of inappropriate therapy, e.g., nitrates should be avoided in RV infarction and vasoconstrictors not as the first line to correct hypotension in RV infarction [11]. Our patient had an uneventful recovery following his RVMI and was discharged 4 days after admission. He preferred to continue medical therapy for his proximal LAD in-stent restenosis.

### Learning point for clinicians

Dome-shaped anterior ST elevation that is most prominent over V1 (as opposed to V2/V3 in LAD occlusion) suggests RV infarction due to RCA occlusion proximal to its RV wall branch. These changes are more profound when the RCA is non-dominant, resulting in a smaller degree of reciprocal anterior ST depression and hence more evident ST elevation in V1.

Interpretation of the ECG using vector concepts is important. When the mean ST segment vector is directed inferiorly, to the right and anteriorly, one should be concerned that RV infarction may be present. ST depression in lead I in this case had indicated a rightward mean spatial ST segment vector, pointing to RV rather than LV infarction.

## Data Availability

Not applicable.
